# On a Substance Found in the Brain and Spinal Marrow of Man, Presenting the Chemical Reaction of Cellulose

**Published:** 1854-05

**Authors:** R. Virchow


### *On a Substance found in the Brain and Spinal Marrow of Man, presenting the Chemical Reaction of Cellulose.* By R. Virchow. (Archiv. f. Path. Anat., VI. 1.)

(Translated for the Examiner.)

It is well known that Charles Schmidt was the first who noticed in the Ascidians, as a constituent of animal tissue, the "cellulose" which previously had only been observed in plants. The researches of Kölliker, Lowig, Schacht, and Huxley soon confirmed this important discovery. Thus, although cellulose was proved to exist in the animal kingdom, its occurrence was confined to but comparatively a low class of the invertebrated animals, and the further discovery, which Gottlieb made in the Euglena viridis, viz., that this infusorium contained paramylon, a substance isomeric with starch, did not much advance our knowledge of the prevalence of the cellulose, as it also had reference to a creature from the lowest classes of the animal kingdom. In the vertebrated animals nothing similar had been met with, and it needed the discovery of Bernard, that the liver generated sugar, to remind us of the fact that perhaps the starchy substances, too, might have their representatives in the higher classes of the animal kingdom.

In a histological point of view it has often occurred to me that the umbilical cord in man possessed a great similarity in its structure to the cellulose in the Ascidians. (See Wurzb. Verhandlungen, 1851, Vol. ii. p. 161, note.) And I was the more confirmed in this idea by Schacht's observation, so that ever since my researches have been more carefully directed to this subject. But in most instances I searched in vain, as in the ova of amphibia and fishes, the peculiar yelk-plates of which I described. (Zertschrift fur Wiss. Zoologic, 1852, Vol. iv. p. 240.) I was more successful, however, recently, when I directed my attention to the so-called "corpora amylacea"* of the brain, of the exact nature of which, as compared with the other starch-like bodies in man, I had not been able to form any very definite conclusions. I soon found that on the application of iodine they

* These corpora amylacea are small yellowish bodies, not unlike starch granules. They were first mentioned by Purkinje, and are found mainly in the walls of the ventricles and medullary substance of the cord.—Tr.

assumed a bluish tint, and in subsequently adding sulphuric acid, the exquisite violet color, which is known to belong to cellulose, and which appeared the more intense as it formed a distinct contrast to the yellow or brown nitrogenous substances around it. I have so frequently repeated these investigations, and with so many precautions, that I consider the results as perfectly certain. For I have instituted comparative researches not only in different human bodies, and in the most different situations, but I have allowed the reagents employed to act under all possible conditions. The best way to proceed is to follow the plan pursued by Mulder and Harting for vegetable cellulose, (see Moleschott's Physiologie des Stoffwechels, p. 103,) which consists in adding first a hydrated solution of iodine, and subsequently dilute sulphuric acid. The solution of iodine must not be too strong, as otherwise we are much impeded by the precipitation of the iodine; but great care must be exerted in allowing it to act properly on the substance in question. This action is generally a very unequal one, on account of the volatility of the iodine, and its great affinity for animal substances, so that it frequently happens that the edge of the object is colored and not the centre, or that in two continuous spots one is penetrated by the iodine, the other not. It is, therefore, always advisable to repeat the application of the iodine several times, without adding too much at once. If the action has been too powerful, the subsequent addition of sulphuric acid will produce a dark reddish-brown color. The most certain manner to proceed is to allow the sulphuric acid to act very slowly. The most beautiful preparations, indeed, I obtained by adding a drop of sulphuric acid to the edge of the thin glass covering a preparation, and allowing it then to remain undisturbed from twelve to twenty-four hours. I have under these circumstances been sometimes able to see the most beautiful light violet blue produced. I think it, finally, necessary to mention that an accidental admixture of starch or cellulose might frequently happen, as little fibres or particles might remain adherent from the cloths with which the glasses are cleaned, which would react in the above described manner. Every precaution having been taken, the following results may be obtained:—

1. The corpora amylacea are chemically different from the concentric spherical corpuscles of which the brain sand is composed, and with which they have hitherto been confounded. The organic basis of these brain-sand granules is evidently nitrogenous: iodine and sulphuric acid color it an intense yellow. The same is true not only of the sandy matter in the pineal gland and the choroid plexuses, but also of that of the Pacchionian granulations, and of the dura mater, as well as of the dentated plates in the spinal arachnoid. In all these parts, except in a few spots in the pineal gland, I have never obtained the characteristic blue reaction. It would, therefore, be henceforth advisable to restrict the term "corpora amylacea" to these cellulose corpuscles.

2. These cellulose corpuscles are found as far as my present researches go, only in the substance of the ependyma* of the ventricle and its prolongations. In this I include the lining of the ventricles of the brain, and the transparent substance in the spinal marrow, described by Kölliker as the "substantia grisea centralis." As regards the ventricles of the brain, I have repeatedly stated that I have found them lined with a membrane belonging to the class of fibrous tissue, and covered with an epithelium. This membrane contains fine cellular elements and a basis substance sometimes of a firm, sometimes of a softer consistence, which is prolonged inwards between the nervous elements without any distinct limitation. In the deepest layers of this membrane, and in close proximity to the nerve fibres, these cellulose granules are most frequently met with, and there again more especially where the ependyma is thickened. They are, therefore, very numerous on the septum lucidum, fornix, stria cornea, and in the fourth ventricle. In the spinal cord the substance which corresponds to the ependyma lies in the middle of the gray mass, in the exact situation where the spinal canal exists in the fœtus. It forms here evidently a rudiment of the obliterated canal, similar to the obliteration of the posterior cornua of the lateral ventricles, so frequently met with. In transverse sections of the cord this substance is easily recognizable as a gelatinous, somewhat resistant, but easily isolated mass, the cells

* The term "ependyma" is used to designate that portion of the lining of the ventricles, of the anterior and posterior cornua, which is not a continuation of the pia mater.—Tr.

of which are much larger and more perfect than those of the cerebral ependyma. But as these cells have been already accurately described by Kölliker, I need not here mention them further. This "spinal ependyma" forms a continuous gelatinous filament, which extends to the *filum terminale*, and might, therefore, be more suitably described as the *central ependyma* filament. In it, also, we find cellulose granules, but more frequently, it seems, in the upper than in the lower portion. In all other situations I have as yet looked for these cellulose granules in vain, and in particular have I been unable to detect them in the cortical layer of the brain, or in the interior of the cerebral substance.

3. As from the experiments of Cl. Bernard, who produced sugar in the urine of a rabbit by wounding the fourth ventricle, there was reason to suppose that this fact might be connected with the existence of cellulose, I examined the brain of the rabbit carefully, but in vain. I found here in the fourth, as well as in the third and in the lateral ventricles, a beautiful pavement epithelium with long cilia, but no cellulose.

4. The cellulose granules seem hence to be connected with the presence of the ependyma substance in certain quantities, and might not improperly be considered as a part of it. But how they are produced from it, it was impossible to recognise. They are unusually minute, scarcely corresponding in size to the nuclei of the ependyma. Can they originate from these? The larger they are the more distinctly laminated they appear. But they do not exhibit anywhere a nitrogenous admixture, distinguishable by its yellow color. Their centre only is generally of a darker blue, and hence, perhaps, denser than their border.

5. The supposition of these bodies being introduced from without is the less probable, because a similar substance is nowhere else known. The cellulose in plants exhibits a number of varieties, but this animal cellulose is distinguishable above all by its slight resistance towards reagents; for concentrated acids and alkalies act on it more powerfully than on vegetable cellulose.

6. In the child I have searched for this animal cellulose in vain, so that, like the brain-sand, it seems to appear in a later stage of development, and may, therefore, perhaps have a pathological import.

Since writing the above, I have repeated and confirmed these observations, and have ascertained in addition, that similar bodies exist in the higher nerves of sense. I found them most abundant in the soft grayish interstitial substance of the olfactory nerve, less frequent in the acoustic, although Meissner's observation (vide Zeitsch. f. Nat. Med. N. F., Vol. iii. p. 358, 363,) would seem to indicate in their situation a proportionately great disposition to their formation. In the optic nerve, Rokitansky appears already to have observed them, and Kölliker informs me, in a novel communication, that he has met with them in the retina. As above-mentioned, the ependyma is prolonged without distinct boundaries between the nervous elements. In the higher nerves of sense, too, I found a continuous extension of a similar substance in the interior of these nerves. This fact, taken in connection with a number of pathological observations, the details of which will be given another time, compels me to infer that a soft substance, in the main belonging to fibrous-tissue, transverses and connects everywhere the nervous elements in the centres, whilst the ependyma is only the free portion extending over the surface of the nervous elements. The opinion that the epithelium of the ventricles is situated immediately on the nervous elements, seems to have been based on a confusion of the interstitial substance with the nerve-substance proper.

I have as yet not been able to isolate the corpora amylacea in sufficient numbers to subject them to a chemical analysis, yet no doubt can possibly be entertained as to their cellulose nature. No other substance is known which reacts in the same manner, and although I have tested the most various animal tissues, and examined all the other concentric bodies that I have met with lately, (as those in the thymus gland and in tumors,) I have found nothing similar. Although it would be very desirable to have a direct proof by analysis, that these bodies contain no nitrogen, we can yet, even without further evidence, consider their analogy with the vegetable cellulose as a settled fact.

(To be continued.)

